# Field-validated microbial deodorant reduces hazardous gas exposure and improves air quality in rural household toilets

**DOI:** 10.1093/jimb/kuag024

**Published:** 2026-10-14

**Authors:** Shangyi Shu, Yujun Shen, Lage Wuri, Yiman Jia, Ao Li, Jingtao Ding, Huihui Wang

**Affiliations:** Academy of Agricultural Planning and Engineering (AAPE), MARA, Beijing, China; Academy of Agricultural Planning and Engineering (AAPE), MARA, Beijing, China; Academy of Agricultural Planning and Engineering (AAPE), MARA, Beijing, China; Academy of Agricultural Planning and Engineering (AAPE), MARA, Beijing, China; State Environmental Protection Key Laboratory of Ecological Effect and Risk Assessment of Chemicals, Chinese Research Academy of Environmental Sciences, Beijing, China; Academy of Agricultural Planning and Engineering (AAPE), MARA, Beijing, China; Academy of Agricultural Planning and Engineering (AAPE), MARA, Beijing, China

**Keywords:** rural sanitation, hazardous gas exposure, microbial intervention, long-term field trial, public health, metagenomics

## Abstract

Malodorous emissions from rural sanitation systems pose a direct environmental health concern. This study developed a composite microbial deodorant (CMD) using five bacterial strains isolated from activated sludge, combined in an optimal 1:1:1:1:1 ratio determined by orthogonal testing, to mitigate emissions of NH₃ and H₂S—key hazardous and odorous pollutants. In laboratory simulations, the CMD reduced NH₃ and H₂S by 82.1% and 86.1%, respectively. A pivotal 6-month field evaluation in three common types of rural household toilets demonstrated sustained, real-world efficacy, with peak removal rates of 79.43% for NH₃ and 80.83% for H₂S. The intervention also conserved nitrogen, reducing total nitrogen loss by 14.8%. Metagenomic analysis revealed the mechanistic basis: the CMD reshaped microbial metabolic pathways by upregulating genes for nitrification (*hao*) and sulfur oxidation (*sqr*), while suppressing reductive genes (*nif, aprA*), thus curbing emissions at source. This work provides robust field evidence that an *in situ* microbial strategy can effectively and sustainably improve household air quality in rural settings, offering a practical, low–cost, and eco–friendly solution for *in situ* odor control in rural sanitation- a critical step for sustaining the health benefits of the ‘‘Toilet Revolution’’ and improving rural living environments.

**One-Sentence Summary** A composite microbial deodorant effectively reduces ammonia and hydrogen sulfide emissions and improves air quality in rural household toilets through 6-month field validation, providing an eco-friendly and low-cost odor control solution for rural sanitation.

## Graphical Abstract

**Graphical Abstract:**
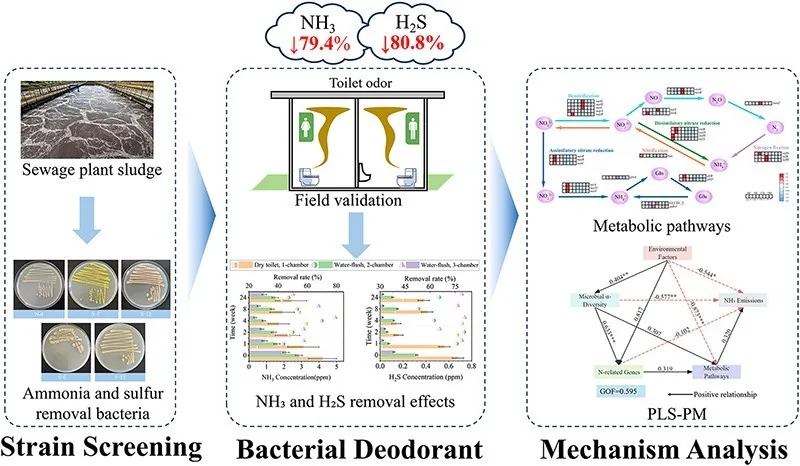

## Introduction

Improving sanitation conditions in rural areas constitutes a critical step toward achieving Sustainable Development Goal 6 and ensuring environmental health equity (Li et al., 2021a). In China, the nationwide ‘‘Toilet Revolution’’ has significantly increased the coverage of sanitary toilets, with coverage reaching ~77% by the end of 2025, addressing a fundamental infrastructure gap (Zheng et al., 2018). However, the sustainability and user acceptance of these systems are often compromised by a persistent secondary problem: the substantial release of volatile malodorous compounds, primarily NH₃ and H₂S, during the storage and treatment of fecal waste (Karnachuk et al., 2021; Li et al., 2024b; Lyu et al., 2024; Ma et al., 2021b).

It is estimated that China produces approximately 0.8 to 0.9 billion tons of fecal matter annually (Rose et al., 2015; Kinhoun et al., 2022). When inadequately decomposed, this waste not only releases malodorous gases but may also harbor pathogenic microorganisms and parasite eggs, creating a combined biological and chemical pollution risk (Papadakis et al., 2000; Rappert and Müller, 2005). The persistent emission of NH₃ and H₂S leads to continuous olfactory nuisance and is associated with health risks such as headaches, anxiety, and respiratory irritation (Papadakis et al., 2000; Rappert and Müller, 2005). In the confined spaces of rural toilet enclosures, these gases create a direct and prolonged exposure pathway for households, posing tangible health risks and discouraging the consistent use of improved facilities, thereby undermining the public health goals of the entire sanitation initiative. Effective odor control is thus not only a technical issue but a public health and behavioral imperative for sustaining sanitation gains.

Current deodorization strategies, such as physical adsorption (Le-Minh et al., 2018; Tung et al., 2009), chemical masking (Syrchina et al., 2023; Wang et al., 2023), or centralized biofiltration (Liang and Qin, 2025), are often ill-suited for decentralized, resource-limited rural settings due to high operational costs, maintenance complexity, or the generation of secondary pollution (Li et al., 2024a, 2024b). Biological approaches using microbial agents offer a promising, eco-friendly alternative by transforming pollutants *in situ* (Barbusinski et al., 2017). While research on microbial deodorants has progressed for livestock manure and industrial waste, their application to human excreta in decentralized toilet systems remains scant (Liu et al., 2023; Lyu et al., 2024). The microbial ecosystem and substrate composition of human feces differ significantly; due to complex dietary structures, the nitrogenous and sulfurous compounds produced during its fermentation are more diverse, resulting in more pungent and persistent odors that present a greater deodorization challenge (Sato et al., 2002; Zhu et al., 2021). This necessitates targeted consortium development. Activated sludge from wastewater treatment plants, with its long–term acclimation to complex organic and nitrogen/sulfur compounds, harbors a rich diversity of microorganisms possessing high metabolic activity and robust stress tolerance, making it an ideal source for isolating potent ammonia– and sulfide–oxidizing strains. Crucially, most existing studies are confined to laboratory simulations, and robust, long-term field validation of microbial agents in real-world rural sanitation contexts is critically lacking. Moreover, from a microbial ecology perspective, effective deodorization is not merely about adding functional strains; it requires a systematic understanding of how introduced consortia reshape the *in situ* microbial ecosystem to redirect the biogeochemical cycling of nitrogen and sulfur. Current research largely lacks a multilevel framework linking ‘‘community assembly—functional gene networks—metabolic fluxes—gaseous emissions’’ (Kuypers et al., 2018; Lawson et al., 2019). This gap between empirical efficacy and mechanistic insight hinders the translation of bioaugmentation strategies into predictable and robust management solutions.

To bridge this gap, this study was designed with a clear public health application perspective. We hypothesized that a specifically constructed microbial consortium could not only degrade NH₃ and H₂S efficiently in controlled conditions but, more importantly, could remain functionally active or induce a sustained functional shift in diverse rural toilet environments, leading to long-term reductions in hazardous gas concentrations. To test this, we (1) isolated and assembled a novel composite microbial deodorant (CMD) from environmental sources; (2) evaluated its deodorizing performance and nutrient conservation effects in laboratory simulations; and (3) conducted a pivotal 6-month field validation across three predominant types of rural household toilets to assess its real-world, sustained efficacy. Furthermore, we employed metagenomic sequencing to elucidate the mechanistic shifts in microbial nitrogen and sulfur metabolic pathways underpinning the observed gas reduction. By integrating community profiling, functional gene analysis, and multivariate statistical modeling, we aim to unravel the multilevel mechanisms underpinning this field–validated biological technology, providing both mechanistic understanding and practical guidance for technology implementation in decentralized sanitation systems.

## Materials and methods

### Isolation and identification of bacterial strains

#### Isolation and preliminary screening

Activated sludge from a Beijing municipal WWTP was enriched in ammonia- and sulfur-targeted media to select strains associated with the target pathways, followed by serial dilutions, plating on selective agar, and colony purification. Seed cultures were prepared and inoculated into ammonia- and sulfur-removal screening media at 30°C; NH_4_^+^-N and S^2−^ removal were quantified using the Nessler and PADA methods, respectively, with appropriate controls and triplicates (Rice et al., 2012; Sharma et al., 2021). Detailed media compositions, enrichment/shaking conditions, plating procedures, and analytical steps are provided in Supplementary Method S1.1.

#### Secondary screening under gas-release simulations

To validate deodorization of volatile gases, NH_3_ and H_2_S release simulations were performed. NH_3_ was captured using a sponge-based collector within a sealed PVC column, and H_2_S was trapped by an absorption solution in a sealed beaker configuration. Concentrations were measured by the sodium hypochlorite–salicylic acid method (for NH_4_^+^) and the methylene blue spectrophotometric method (for H_2_S) (Bower and Holm-Hansen, 1980; Kourtidis et al., 2008). Device structure, dimensions, replacement schedules, extraction, and quantification steps are described in Supplementary Method S1.2 and Supplementary Figure S1.

#### Strain identification and growth characterization

High-performing strains were identified using standard morphological, physiological/biochemical, and molecular methods. 16S rRNA gene sequencing was aligned against EzBioCloud; phylogenetic trees were constructed in MEGA 11 with bootstrap evaluation; growth curves were obtained via OD_600_. Expanded identification procedures, biochemical panels and antagonism outcomes are reported in Supplementary Method S1.3, Supplementary Table S1 and S2, and Supplementary Figures S2−S4.

### Formulation and laboratory evaluation of the CMD

An orthogonal experimental design (L_8_(2⁵)) was used to optimize the consortium ratio, with the five strains as factors. The optimal combination was defined as the CMD. The specific orthogonal design setup, strain codes, mixing logic, and validation experiment details (container dimensions, sampling protocols) are detailed in Supplementary Method S1.4.

For lab-scale evaluation, the CMD was inoculated at 10% (v/w) into a simulated fecal matrix (200 g) within a sealed reactor. A control (CK) received an equal volume of sterile medium. The reactors were maintained at ambient temperature (25 ± 2°C). Headspace concentrations of NH₃ and H₂S were monitored daily. Samples were periodically collected for analysis of pH, NH₄⁺-N, NO₃⁻-N, TN, and SO₄²⁻ using standard methods (Gustafsson, 1960; Rice et al., 2012).

### Field application trial in rural household toilets

A 6-month field validation was conducted in a rural village in Hebei Province, China. Three dominant types of household sanitation facilities were included: (1) dry toilet with a single storage pit, (2) pour-flush toilet with a double-vault septic tank, and (3) pour-flush toilet with a triple-tank septic tank. Five representative households were selected for each type.

The CMD was prepared as per the optimal ratio, cultured to late-exponential phase, concentrated, and resuspended in sterile saline. For each system, 2.0 L of the CMD suspension was applied to the first pit/tank at the start (Week 0), followed by a supplemental dose of 1.0 L at Week 4. Ambient concentrations of NH₃ and H₂S at the toilet enclosures were monitored at Weeks 1, 2, 4, 8, and 24 using portable detectors and absorption methods, identical to laboratory protocols.

### Metagenomic sequencing and bioinformatic analysis

Total genomic DNA was extracted from fecal samples (CK and CMD-treated) collected during the lab-scale experiment using the E.Z.N.A.® Soil DNA Kit. DNA quality and concentration were verified. Paired-end libraries were constructed and sequenced on an Illumina Nextseq 2000 platform.

Raw reads were processed using fastp for quality control. De novo assembly was performed with MEGAHIT. Gene prediction, construction of a nonredundant gene catalog, functional annotation against the KEGG database, and gene abundance profiling were conducted using a standardized pipeline as fully detailed in Supplementary Method S1.5.

### Statistical analysis

All experimental data are presented as mean ± standard deviation (SD). Statistical significance of differences between treatment and control groups was assessed using Student’s t-test or one-way ANOVA followed by Tukey’s post-hoc test in OriginPro 2024, with a significance threshold of *p* < .05.

## Results and discussion

### Construction and functional validation of the deodorizing consortium

A total of 29 strains were isolated after enrichment on sulfur- and ammonia-targeted media, including 16 ammonia-removing (N-1–N-16) and 13 sulfur-removing (S-1–S-13) isolates. In the preliminary screening, NH₄⁺-N and S²⁻ removal efficiencies varied widely (Figure 1A and B). Among ammonia–removing strains, six isolates (N–4, N–7, N–9, N–10, N–11, and N–12) exhibited the highest NH₄⁺–N removal (93.60%, 70.47%, 66.70%, 67.03%, 68.62%, and 72.57%, respectively); based on these results, N–4, N–7, and N–12 were selected for further evaluation. For sulfur–removing strains, eight strains exceeded 90% S²⁻ removal within 8 h; S–2 (99.56%), S–11 (99.49%), S–13 (99.28%), and S–6 (98.66%) were shortlisted (Figure 1B). During secondary screening in simulated fecal matrices (Figure 1C and F), NH₃ emissions declined over time in all treatments and were consistently lower than the control during the first 5 days. The ranking of cumulative NH₃ emissions (CK > N–11 > N–10 > N–9 > N–8 > N–4 > N–7) corresponded to removal efficiencies of 72.05–87.26%; N–7 and N–4 performed best. For H₂S, emissions peaked within 3 days and then declined; peak values followed the order CK > S–11 > S–2 > S–13 > S–6. Final H₂S removal reached 85.20% (S–6) and 81.58% (S–13); therefore S–6 and S–13 were advanced to consortium development.

**Figure 1:**
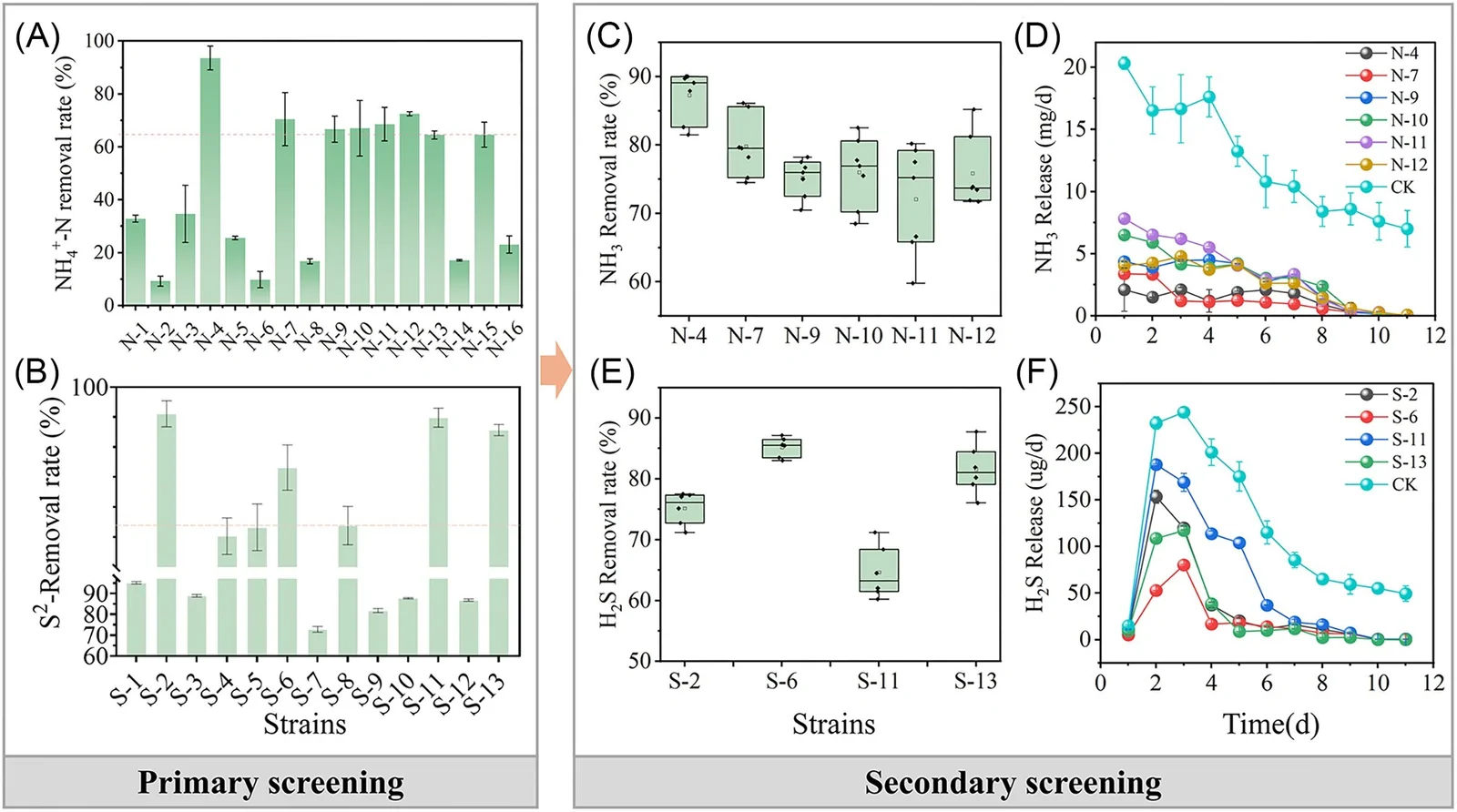
Screening and validation of deodorizing strains. (A) Removal efficiencies of individual isolates for NH₄⁺-N in primary screening. (B) Removal efficiencies of individual isolates for S²⁻ in primary screening. (C) NH₃ removal efficiencies of shortlisted ammonia-removing strains under secondary screening. (D) Time-course NH₃ release in simulated fecal matrices. (E) H₂S removal efficiencies of shortlisted sulfur-removing strains under secondary screening. (F) Time-course H₂S release in simulated fecal matrices. Error bars denote mean ± SD (*n* = 3). CK, sterile control.

Colony morphology, physiological-biochemical traits, growth curves, and antagonism matrices for the five dominant strains (N-4, N-7, N-12, S-6, and S-13) are provided in Supplementary Results (Figures S2–S4, Table S1–S2). Phylogenetic trees derived from 16S rRNA sequencing are shown in Supplementary Figure S3. Phylogenetic analysis confirmed their close relationship with known type strains (Figure S3). Supplementary Table S3 reports the 16S rDNA alignment results; the five selected strains matched most closely to *Alcaligenes faecalis* subsp. phenolicus (N-4, 99.86%), *Arthrobacter luteolus* (N-7, 99.57%), *Lysinibacillus fusiformis* (N-12, 99.93%), *Stutzerimonas balearica* (S-6, 99.71%), and *Pseudomonas guariconensis* (S-13, 99.43%). The functional attributes of these strains, as documented in previous studies, provide a strong basis for their synergistic action in a deodorizing consortium. Strains of the genus possess both nitrification and denitrification capabilities (Chen et al., 2021; Neerackal et al., 2016; Rehfuss and Urban, 2005; Zhang et al., 2022). For instance, *Alcaligenes faecalis* subsp. phenolicus, isolated by Regar et al. (2023) from river water in India, was found to degrade indole efficiently. Moreover, *Alcaligenes faecalis* strain S1 has been reported to exhibit sulfur-oxidizing activity (Song et al., 2019). Strains of the genus Arthrobacter have been widely used in environmental remediation, particularly for the biodegradation of antibiotics (Deng et al., 2018) and pesticides (Cao et al., 2021; Qian et al., 2024). However, there are currently no reports on the role of *Arthrobacter luteolus* in removing malodorous gases. *Lysinibacillus fusiformis* is known to produce enzymes capable of degrading complex organic pollutants, including the breakdown of persistent polycyclic aromatic hydrocarbons into less toxic compounds. It also participates in denitrification, converting nitrates into nitrogen gas, and thereby plays a crucial role in the nitrogen cycle (Gupta et al., 2024). The genus Pseudomonas has been extensively studied and applied in agriculture (Zia et al., 2021), the removal of environmental pollutants (Gan et al., 2024; Silva et al., 2019), and medicine (Li et al., 2021b). Notably, *Stutzerimonas* balearica, previously classified within Pseudomonas, functions as an aerobic nitrifying bacterium. It has been reported to effectively participate in the nitrification process under conditions with a carbon-to-nitrogen (C/N) ratio of 5.0–7.5, thereby reducing NH₃ emissions (Zhang et al., 2023). Collectively, these strains exhibit diverse advantages in nitrogen and sulfur metabolism, pollutant degradation, and environmental adaptability.

Using the five dominant strains identified (N-4, N-7, N-12, S-6, S-13), we implemented a two-level, five-factor L₁₆(2⁵) orthogonal design (16 combinations) to evaluate NH₃ and H₂S removal (Table 1). As shown in Figure 2, strain effects depended on the combination. Combination A—an equal–ratio mixture of all five strains (1:1:1:1:1)—consistently performed best, achieving 82.1% NH₃ and 86.1% H₂S removal, which was significantly higher than other combinations (*p* < .05; Figure 2C). Combination A was therefore selected for validation.

**Figure 2:**
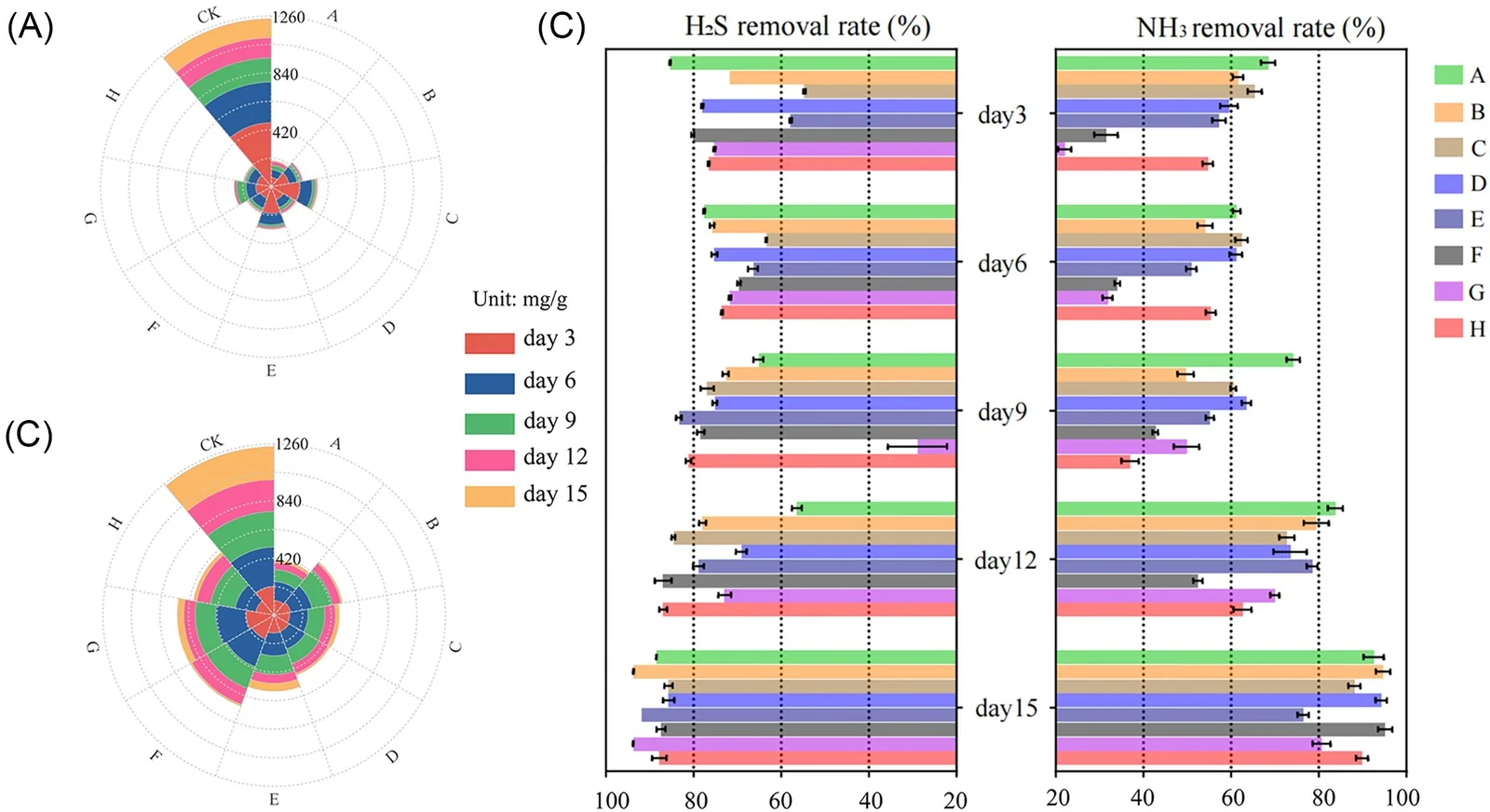
Effects of strain combinations on gas emissions and deodorization: (A) NH₃ emission trends; (B) H₂S emission trends; (C) NH₃ and H₂S removal efficiencies across combinations.

**Table 1:** Factor and level assignment table L8(2⁵) for orthogonal testing of deodorizing strain combinations.

Treatment	N-4	N-7	N-12	S-6	S-13
A	+	+	+	+	+
B	+	+	–	–	–
C	+	–	+	–	–
D	+	–	–	+	+
E	–	+	+	–	+
F	–	+	–	+	–
G	–	–	+	+	–
H	–	–	–	–	+

Note: ‘‘+’’ indicates strain inoculation; ‘‘–’’ indicates no inoculation.

While the overall deodorization performance of the consortium was comparable to that of some individual strains, its core advantage lies in its adaptability and functional complementarity, which significantly enhances its practical applicability. During dynamic deodorization processes, single strains typically perform well under specific optimal growth conditions. However, when environmental parameters deviate from these conditions, their activity rapidly declines, resulting in unstable or short–lived deodorization effects. In contrast, microbial consortia exhibit a ‘‘functional relay’’ mechanism, in which different strains dominate at different stages, ensuring continuous and efficient deodorization by maintaining active functional populations throughout the entire process (Wang et al., 2022b; Xin et al., 2016). Moreover, the complexity of fecal odor compounds makes it challenging for a single strain to remove multiple gases effectively. For example, Ma et al. (2021a) found that *Bacillus licheniformis* achieved a high H₂S removal rate of 99.3%, but its NH₃ removal capacity was limited. In microbial consortia, synergistic interactions between strains enable improved functionality. Studies have shown that aerobic decomposers can rapidly degrade complex organic matter into smaller intermediate compounds, which can then serve as carbon and energy sources for nitrifying or sulfur–oxidizing bacteria (Lawson et al., 2019). This synergy enhances the overall stability and long–term performance of the microbial consortium (Wang et al., 2025a). Compared to recent studies, the removal efficiencies of our CMD (82.1% for NH₃ and 86.1% for H₂S) are comparable to those reported by Lyu et al. (2024) using a composite agent for toilet fecal odor, while our consortium exhibits greater phylogenetic diversity, integrating ammonia–oxidizing, sulfur–oxidizing, and organic–degrading functionalities. This functional complementarity positions the CMD as a robust agent for complex waste matrices.

For validation, Combination A (treatment, T) was inoculated into feces; an equal volume of sterile medium served as control (CK). NH₃ and H₂S were monitored throughout. While the use of a sterile control effectively demonstrated the net effect of the CMD over indigenous microbiota, future studies incorporating positive controls (e.g., commercial deodorants) would be valuable for benchmarking its relative performance. NH₃ increased during the first 5 days in both groups, peaking at 436 ppm (CK) vs. 248.7 ppm (T) (Figure 3A). This initial surge in NH₃ release may be attributed to the high content of nitrogen-rich organic compounds such as proteins and amino acids in feces, which serve as abundant substrates for indigenous microorganisms. These microbes likely proliferated rapidly and intensified ammonification, leading to elevated NH₃ levels in the CK group (Hou et al., 2023). In contrast, the microbial consortium inoculated in the treatment group may have suppressed NH₃ production through the following mechanisms: (1) inhibition of indigenous microbial degradation of organic matter; (2) competition with native ammonia-producing microbes for limited nutrients and ecological niches, thereby limiting their overgrowth; and (3) rapid utilization of readily degradable carbon sources by the exogenous strains, altering microbial succession and reducing the intensity of ammonification-driven metabolism in the early phase (Li et al., 2024c; Zhang et al., 2024). From day 6 onward, NH₃ emissions began to decline, possibly due to the depletion of available organic substrates or the accumulation of metabolic by-products exerting toxic effects on ammonia-producing microbes. The microbial consortium in the treatment group may have facilitated the transformation of organic nitrogen into nitrate or other assimilable forms, thereby reducing NH₃ generation (Zhao et al., 2024). Over time, NH₃ emissions reached a relatively stable state, suggesting that nitrogen transformation processes in the fecal matrix had reached equilibrium (Bernal et al., 2009). Notably, NH₃ levels in the treatment group remained consistently lower than in the control group throughout the experiment. This sustained reduction (*p* < .05 at multiple time points) strongly indicates that the microbial consortium did not merely cause a temporary shift in the microbial community structure, but rather established a stable, functionally effective microbial population that supports ammonia suppression through enhanced nitrogen transformation pathways (Liu et al., 2024). Functional microbes such as nitrifiers and assimilatory nitrogen-utilizing bacteria likely occupied dominant ecological niches and continuously contributed to nitrogen conversion. These results highlight the long-term potential of the microbial deodorizing agent in suppressing NH₃ emissions.

**Figure 3:**
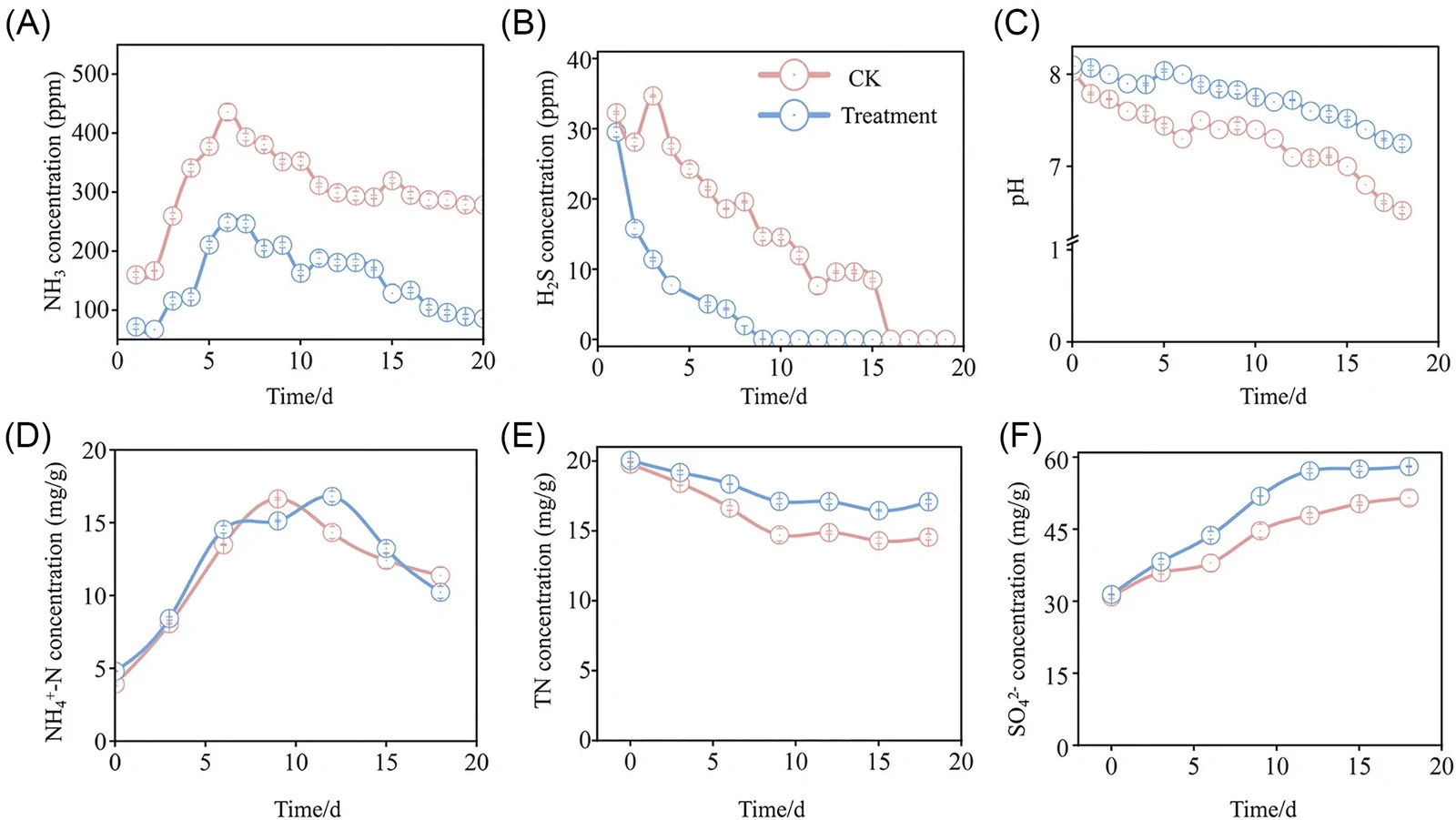
Dynamics during consortium deodorization: (A) NH₃ emission; (B) H₂S emission; (C) pH; (D) NH₄⁺-N; (E) TN; (F) SO₄²⁻.

The dynamics of H₂S release during the deodorization process are shown in Figure 3B. H₂S is typically produced by sulfate-reducing bacteria (SRB) under anaerobic conditions through the decomposition of sulfur-containing organic matter. In this study, the H₂S emission level in the treatment group (T) consistently remained lower than that in the control group (CK) and declined to near the detection limit by day 8. This rapid and effective suppression of H₂S emissions demonstrates the consortium’s strong sulfur–oxidizing capacity. Several mechanisms may explain this phenomenon: (1) The microbial consortium likely contained a high proportion of aerobic bacteria such as *Pseudomonas* spp., which are known for their active respiration and potential to produce extracellular polymeric substances (EPS). These EPS can improve the porosity and aeration of the fecal matrix (Wang et al., 2021). In addition, aerobic biofilms or microcolonies formed on the surface or within fecal particles can consume diffused oxygen, thereby expanding aerobic zones within the substrate. This shift toward microaerobic conditions inhibits the activity of strictly anaerobic SRB (Gao and Fan, 2023). (2) Competition and antagonism among microbial populations. The aerobic and facultatively anaerobic bacteria in the consortium compete directly with SRB for nutrients and spatial niches. The rapid utilization of readily degradable organic matter by the highly active exogenous strains may reduce the availability of electron donors for SRB, thereby indirectly inhibiting the sulfate reduction pathway and H₂S production (Wang et al., 2022b).

The ecological significance of these findings extends beyond mere removal efficiencies. The significantly lower NH₃ and H₂S emissions in the treatment group (*p* < .01) reflect a fundamental shift in the balance of nitrogen and sulfur cycling within the fecal matrix—from reductive, gas–producing pathways toward oxidative, immobilizing pathways. This shift, orchestrated by the introduced consortium, demonstrates the feasibility of ‘‘microbial ecosystem engineering’’ to redirect elemental fluxes and mitigate pollutant release at source.

PH is a critical factor influencing microbial activity. As shown in Figure 3C, both the treatment and control groups exhibited a similar trend in pH variation: an initial decrease, followed by a slight increase, and then a subsequent decline, resulting in an overall downward trend. The initial pH of the control group was 8.1, which steadily dropped to 6.5 by day 18, indicating significant acidification. In contrast, the treatment group showed fewer minor fluctuations, maintaining a pH of 7.25 at the end of the experiment. The early stage of fecal fermentation is typically characterized by acid-producing fermentation, during which malodorous compounds such as H₂S and mercaptans are released. The addition of the microbial consortium appears to mitigate this acidification, possibly due to the buffering effect of its metabolic by-products. Moreover, a neutral to slightly alkaline environment is favorable for the activity of nitrifying bacteria, which facilitates the conversion of NH₄⁺-N to NO₃⁻-N (Liang et al., 2024). Other studies have shown that genes involved in sulfur oxidation pathways are more abundant under alkaline conditions, which may contribute to the reduction of H₂S production and emission [Miao et al., 2024]. The primary microbial processes involved in the nitrogen cycle include ammonification, ammonium assimilation, nitrification, denitrification, anaerobic ammonia oxidation (anammox), and nitrogen fixation (Hoang et al., 2022). As an essential inorganic nitrogen source, NH₄⁺-N plays a key role in microbial metabolism. As shown in Figure 3D, both groups followed a similar trend in NH₄⁺-N dynamics: levels increased in the early phase due to ammonification by indigenous microbes and peaked on day 9 (CK: 16.64 mg/g) and day 11 (T: 16.81 mg/g), indicating extensive decomposition of nitrogen-rich organic matter. After reaching peak levels, NH₄⁺-N concentrations declined in both groups. By the end of the experiment, the control group retained a higher NH₄⁺-N level (11.38 mg/g) compared to the treatment group (10.23 mg/g). This suggests that inoculation with the microbial consortium promoted the rapid growth of nitrifying bacteria, thereby reducing NH₄⁺-N content and enhancing NO₃⁻-N accumulation (Chen et al., 2022). Liang et al. (2024) reported a similar trend in ammonium dynamics during composting of wastewater sludge with microbial inoculants. TN is a key indicator of nitrogen loss. As shown in Figure 3E, most TN loss occurred during the early stage of the experiment due to volatilization of nitrogen-containing gases and biodegradation of nitrogen-rich organics. By the end of the experiment, TN content in the treatment group was 17.09 mg/g, 14.8% higher than that of the control group (14.56 mg/g). This suggests that the inoculated microbial consortium, possessing nitrification capability, facilitated the rapid conversion of NH₃ into other nitrogenous forms, thus reducing its volatilization. Suppressing NH₃ emissions is a critical component of nitrogen conservation (Tian et al., 2023). Therefore, the results of this study indicate that the microbial deodorizing agent effectively reduces nitrogen loss and promotes nitrogen retention by mitigating NH₃ volatilization.

As shown in Figure 3F, the SO₄²⁻ content exhibited a continuous upward trend throughout the experiment. This increase may be attributed to the activity of indigenous sulfur-oxidizing bacteria, which, under localized microaerobic conditions, oxidized part of the H₂S into sulfate. The initial SO₄²⁻ concentration in the control group was 30.75 mg/g, rising to 42.53 mg/g by the end of the experiment. The treatment group started with a similar SO₄²⁻ concentration but reached a higher final level of 47.43 mg/g. When sulfur-containing malodorous gases such as H₂S are released during fecal decomposition, denitrifying sulfur-oxidizing bacteria can convert H₂S into elemental sulfur or sulfate through oxidative processes. In cases where the odor originates from organic sulfur compounds, these must first be degraded by heterotrophic microorganisms into H₂S intermediates, which are then oxidized by denitrifying sulfur-oxidizing bacteria into final products—typically elemental sulfur or sulfate (Wei et al., 2016). A near-neutral pH environment can promote the activity of sulfur-oxidizing bacteria, enhancing the conversion of H₂S to SO₄²⁻, while simultaneously inhibiting the anaerobic metabolism of SRB. This environmental shift helps to suppress the formation and release of H₂S.

### Field application and practical efficacy in rural sanitation systems

To critically assess the real-world applicability and durability of the developed CMD, a 6-month field trial was conducted across three dominant types of rural household sanitation facilities in Hebei Province, China. The systems included a dry toilet with a single storage vault (Type I), a pour-flush toilet with a double-vault septic tank (Type II), and a pour-flush toilet with a triple-tank septic tank (Type III). The CMD demonstrated a capacity for rapid and substantial odor mitigation across all systems, with a consistent temporal dynamic characterized by a swift response followed by peak efficacy around the fourth week post-application (Figure 4). In Types I, II, and III toilets, NH₃ concentrations were reduced from initial levels of 4.2, 2.85, and 2.11 ppm to 1.7, 0.85, and 0.36 ppm at week 4, corresponding to removal efficiencies of 59.52%, 70.18%, and 79.43%, respectively. A similarly pronounced effect was observed for H₂S, with the Type III system achieving a peak reduction from 0.12 ppm to 0.023 ppm, representing an 80.83% removal rate at week 4. This ‘‘rapid response, peak at 1 month’’ pattern indicates a consistent period required for the inoculated consortium to adapt, establish, and reach its maximum functional biomass within the complex fecal matrix, effectively outcompeting indigenous odor-producing microbes.

**Figure 4:**
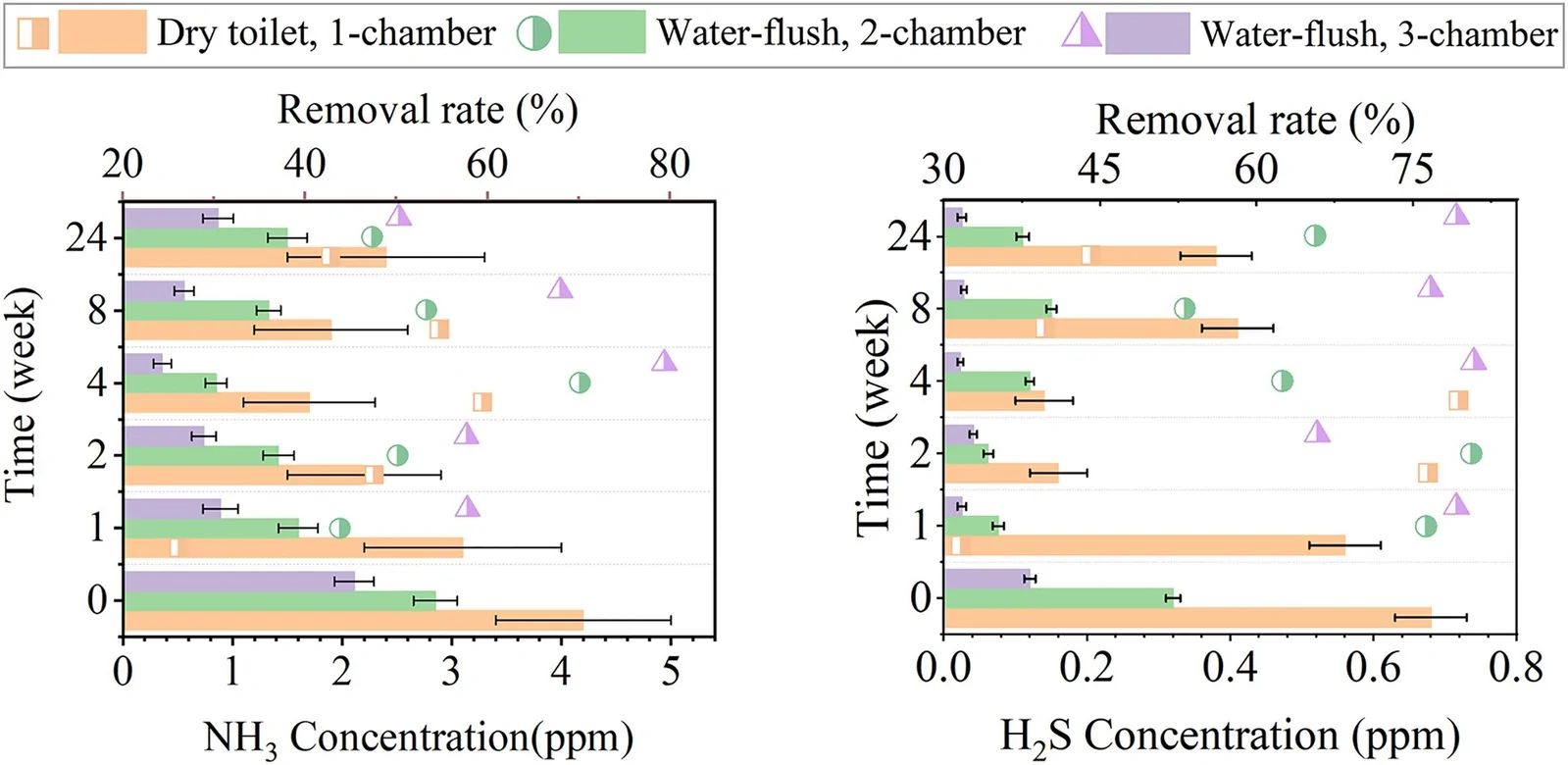
Field performance dynamics of the CMD across three rural sanitation system types over 24 weeks.

A central finding of this field study is the clear dependency of the CMD’s performance and stability on the engineering design of the sanitation infrastructure. The triple-tank system (Type III) exhibited the most superior and stable odor control, particularly for H₂S, where removal rates remained exceptionally high at 76.67% and 79.17% during weeks 8 and 24, respectively. A central finding of this field study is the clear dependency of the CMD’s performance and stability on the engineering design of the sanitation infrastructure. The triple-tank system (Type III) exhibited the most superior and stable odor control, particularly for H₂S, where removal rates remained exceptionally high at 76.67% and 79.17% during weeks 8 and 24, respectively. The superior performance of the Type III triple-tank system may be associated with its compartmentalized structure, which improves hydraulic separation and prolongs substrate–microbe contact compared with less structured systems (García-Avila et al., 2023). This configuration could create more heterogeneous microenvironments and provide favorable niches for facultative aerobic and sulfur-oxidizing microorganisms involved in NH₃ and H₂S mitigation, as sulfur-oxidizing bacteria are widely involved in biological H₂S removal and are sensitive to oxygen availability (Kang et al., 2020). Therefore, infrastructure design may act as an important environmental filter shaping the functional stability of the introduced consortium. Further studies integrating *in situ* redox profiling and microbial population tracking would help clarify the engineering–microbial interactions underlying this enhanced performance. In contrast, the single-vault dry toilet (Type I), characterized by a more static and less structured environment, presented a more challenging niche for the introduced consortium. This system showed greater performance fluctuation, with the H₂S removal rate declining to 39.71% by week 8.

Following the peak at week 4, a gradual attenuation in removal efficiency was observed across all systems; for instance, the NH₃ removal rate in the Type III toilet settled to 50.29% by week 24. However, a finding of paramount importance for practical application is that gaseous emissions in all CMD-treated systems remained consistently and significantly below their preapplication baselines throughout the entire 24-week monitoring period. This confirms that the CMD induced a sustained functional shift in the *in situ* microbial ecosystem rather than a transient perturbation. The gradual decline from peak efficiency likely reflects the consumption of readily available substrates and a dynamic re-equilibration between the inoculated and native microbial populations. Nonetheless, the long-term suppression below baseline levels strongly suggests successful niche modification or lasting metabolic pathway alterations, underscoring the resilience of the intervention’s effect.

From a public health and environmental management perspective, these results translate into actionable strategies. The demonstrated pattern of efficacy provides a scientific basis for implementing periodic supplementary dosing regimens (e.g., a booster dose at week 4 as done in this trial) to maintain optimal performance over extended periods. This field validation thus elevates the CMD from a laboratory proof-of-concept to a verified, practical technology. It offers a feasible biological strategy for sustained *in situ* improvement of household toilet air quality, directly targeting the reduction of resident exposure to hazardous air pollutants (NH₃, H₂S) and contributing to enhanced sanitation acceptability and health outcomes in rural communities.

While these field results are promising, translating this technology from controlled trials to widespread practical application requires addressing several challenges. First, the complex and variable composition of real fecal waste, fluctuating temperatures across seasons, and diverse indigenous microbial communities across different geographical regions may influence the colonization efficiency and functional expression of the CMD. Our ongoing winter-phase monitoring in Hebei Province aims to specifically assess the agent’s adaptability to low-temperature conditions. Second, the economic feasibility of large-scale production, long-term preservation of viable cells, and development of simple, low-cost application protocols are critical determinants for adoption by rural households. Third, a comprehensive ecological safety assessment is necessary to evaluate any long-term impacts of introducing exogenous functional strains on native microbial ecosystems, ensuring that key nutrient cycling processes are not disrupted and that no unintended ecological risks arise. Addressing these challenges through targeted research will be essential for the safe, standardized, and sustainable implementation of this microbial deodorization strategy in rural environmental improvement initiatives.

Although the field trial demonstrated sustained suppression of NH₃ and H₂S emissions over 24 weeks, we did not perform strain-specific qPCR or absolute-abundance tracking of the introduced strains during the field period. Therefore, the observed long-term effect should be interpreted as system-level functional persistence rather than direct evidence of permanent colonization by each exogenous strain. The gradual attenuation after week 4 likely reflects substrate depletion and dynamic re-equilibration between the introduced consortium and indigenous fecal microbiota under strong niche-exclusion pressure. Future studies should combine strain-resolved metagenomic mapping, internal-standard-based absolute metagenomics, and strain-specific qPCR/ddPCR to quantify the persistence of Alcaligenes, Stutzerimonas, and Pseudomonas populations *in situ*.

### Microbial community dynamics in response to the consortium

High-throughput metagenomic sequencing was performed on treatment (T) and control (CK) samples to assess community shifts during deodorization. Rarefaction curves indicated near-saturated sequencing depth (Figure S5), and OTU richness profiles supported adequate coverage (Figure S6). Alpha-diversity indices showed time-dependent changes in both groups (Figure 5A–D). The Chao1 index in CK declined at day 6 and then rose, whereas T remained stable early, increased sharply at day 12, and slightly declined by day 18, overall staying below CK—consistent with selective suppression of some indigenous taxa following inoculation (Daly et al., 2018; Schloss et al., 2009). This pattern suggests that the introduced functional strains successfully colonized and exerted competitive pressure on certain native populations, a key indicator of effective niche establishment. Figure 5B shows that the Shannon index in both groups increased over time, indicating a trend toward more even microbial communities and a decrease in the dominance of a few bacterial taxa (Hill et al., 2003). However, the Shannon index of the T group remained consistently lower than that of the CK group at all time points (*p* < .05 at days 12 and 18), suggesting that the introduced functional strains proliferated rapidly and occupied dominant ecological niches, thereby decreasing community evenness (Delgado-Baquerizo et al., 2018; Zhang et al., 2018). From an ecological perspective, this reduction in evenness reflects successful niche occupation by the inoculated consortium, which is precisely the intended outcome for bioaugmentation—establishing functionally dominant populations capable of redirecting metabolic fluxes. The Simpson index (Figure 5C) generally increased throughout the experiment, with the T group showing significantly higher values than the CK group during the later stages (days 12 and 18, *p* < .05), further supporting the dominance of specific functional bacteria in the microbial community (Simpson, 1949). The Shannoneven index (Figure 5D) followed a similar trend to the Shannon index, indicating an increase in community evenness as the experiment progressed. Overall, the microbial diversity and richness in the treatment group showed a slight decline, while the dominance and ecological niche occupation of specific functional strains were enhanced. These findings are consistent with ecological niche competition theory (Nemergut et al., 2013; Prosser et al., 2007), where introduced species with competitive advantages can reshape community structure by occupying key metabolic niches.

**Figure 5:**
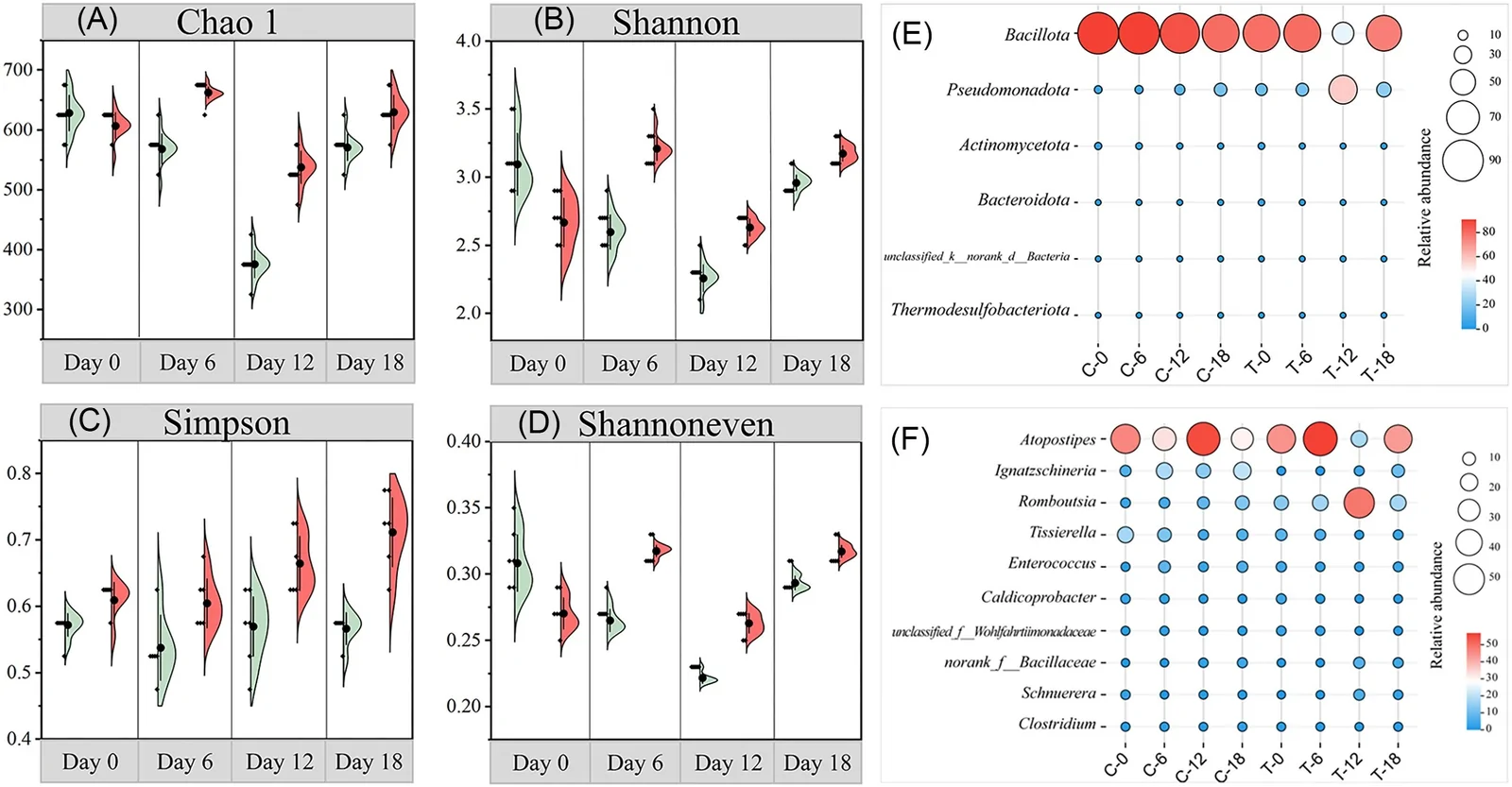
Community responses to the composite microbial agent. (A–D) Alpha-diversity indices (Chao1, Shannon, Simpson, Shannoneven) for treatment (T) and control (CK) across time. (E) Phylum-level community structure (top six phyla). (F) Genus-level community structure (top fifteen genera). Points denote biological replicates; shapes/size encode relative abundance as indicated. See Figures S5 and S6 for rarefaction and OTU richness diagnostics.

To investigate the succession of bacterial communities at different stages of deodorization, bacteria at the phylum and genus levels were analyzed, focusing on the top six most abundant phyla and the top fifteen genera. The results are shown in Figure 5E. Throughout the experimental period, the dominant bacterial phyla included *Bacillota* (formerly *Firmicutes*), *Pseudomonadota, Actinomycetota, Bacteroidota, unclassified_k_norank_d_Bacteria*, and *Thermodesulfobacteriota*.

According to Wang et al. (2022c), *Bacillota, Actinomycetot*a, and *Bacteroidota* are core functional taxa involved in nitrogen transformation processes. These phyla not only participate in nitrification and denitrification but also play crucial roles in organic matter degradation and maintaining microbial community stability, thereby supporting the efficient operation of nitrogen removal systems. *Bacillota*, a significant component of intestinal microbiota, was highly abundant at the beginning of the experiment, with relative abundances of 90.91% (CK) and 79.74% (T). By day 6, relative abundances were 90.90% (CK) and 80.42% (T); by day 12, they had decreased to 86.90% (CK) and 40.76% (T); and by day 18, though reduced from initial levels, *Bacillota* still dominated (75.96%–80.37%). Prior research has shown that *Bacillota* thrives in harsh environments and efficiently degrades organic matter, particularly lignin and cellulose (Wang et al., 2024a), suggesting that its relative abundance is closely linked to changes in organic content. *Pseudomonadota* is commonly associated with denitrification in ecological treatment processes (Wang et al., 2025b). Qianxi found that this phylum enhances the expression of *nirK* and *nosZ* genes, thereby improving denitrification efficiency (Ao et al., 2025). In the CK group, the relative abundance of *Pseudomonadota* showed an increasing trend, reaching 17.81% by day 18. In contrast, the treatment group exhibited a sharp initial increase to a peak of 58.19%, followed by a significant decrease to 22.36%, which remained higher than in the CK group. Notably, *Alcaligenes faecalis, Stutzerimonas balearica*, and *Pseudomonas guariconensis*—all strains used in the deodorizing microbial consortium—belong to *Pseudomonadota*, indicating successful colonization and stimulation of this phylum by the inoculated strains. *Actinomycetota* plays a major role in degrading recalcitrant organic matter and is a key bacterial group involved in lignocellulose decomposition during aerobic composting (Hou et al., 2023). It is also crucial for sulfur oxidation processes (Liu et al., 2022). *Bacteroidota* is considered a group of bacteria capable of degrading organic matter and breaking down complex organics, and has been reported to show a significant negative correlation with NH₃ production during sewage sludge composting (Zheng et al., 2022).

At the phylum level, the addition of the microbial consortium effectively improved the fecal microbial environment by enhancing both the capacity for organic matter degradation and the potential for nitrogen metabolism. As shown in Figure 5F, both groups shared the same dominant bacterial genera but differed in their relative abundances. The genus *Savagea*, commonly found in pig manure, has been reported to be negatively correlated with dimethyl sulfide levels, suggesting its role in reducing sulfur-containing gas emissions (Wang et al., 2024b). In both groups, *Savagea* maintained a relatively high abundance (17.53%–41.04%) throughout the experiment, although a sharp decline was observed in the treatment group on day 12. This may be due to *Savagea*’s ability to utilize sulfur-containing substrates; as H₂S concentrations declined in the later stages of the experiment, the abundance of Savagea also decreased. The genus *Atopostipes* is known to metabolize tryptophan into indole-3-lactic acid, which can be further converted into indole and skatole, major contributors to fecal odor (Cho et al., 2015). On day 18, the relative abundance of *Atopostipes* in the CK group was significantly higher (20.45%) than in the treatment group (9.82%, *p* < .05), indicating that the microbial consortium may have inhibited its growth and thereby reduced the production of odorous compounds. *Ignatzschineria*, a genus within the phylum *Pseudomonadota*, displayed a trend consistent with that of its parent phylum. This suggests that the observed changes may be due to competitive displacement by Alcaligenes or Pseudomonas species introduced through the microbial inoculant. Enterococcus, a genus of facultative pathogenic bacteria, is known for its ability to survive under harsh conditions, including high temperatures and chemical disinfectants. Over the past 30 years, it has become the third most common nosocomial human pathogen (Miller et al., 2014). Throughout the experiment, Enterococcus maintained a higher relative abundance in the CK group than in the treatment group, suggesting that inoculation with the microbial consortium may contribute to the suppression of potentially pathogenic bacteria—an important co-benefit for sanitation safety.

To explore the relationships between physicochemical parameters (pH, TN, SO₄²⁻, NH₄⁺-N, NO₃⁻-N, NH₃ concentration, and H₂S concentration) and bacterial communities during the deodorization process, a Spearman correlation analysis was conducted between the top 15 most abundant bacterial genera and the measured environmental variables. The results are presented in Figure 6A. SO₄²⁻ concentration showed a significant or highly significant positive correlation (*p* < .01 or *p* < .001) with the abundances of *Enterococcus, Romboutsia*, and *Bifidobacterium*, and a significant negative correlation (*p* < .05) with *Ignatzschineria* and *unclassified_f_Wohlfahrtiimonadaceae*. The abundances of *Enterococcus, Romboutsia*, and *Bifidobacterium* were also significantly negatively correlated with H₂S concentrations (*p* < .05), while *Ignatzschineria* showed a significant positive correlation with H₂S levels (*p* < 0.05). This may be due to the role of *Ignatzschineria* in organic matter degradation, which could stimulate the growth of SRB and thereby enhance H₂S production within the compost matrix (Gao et al., 2021). The genus *Atopostipes* was significantly positively correlated with pH and TN (*p* < .05), while *Enterococcus* abundance was significantly positively correlated with NO₃⁻-N concentrations (*p* < .05).

**Figure 6:**
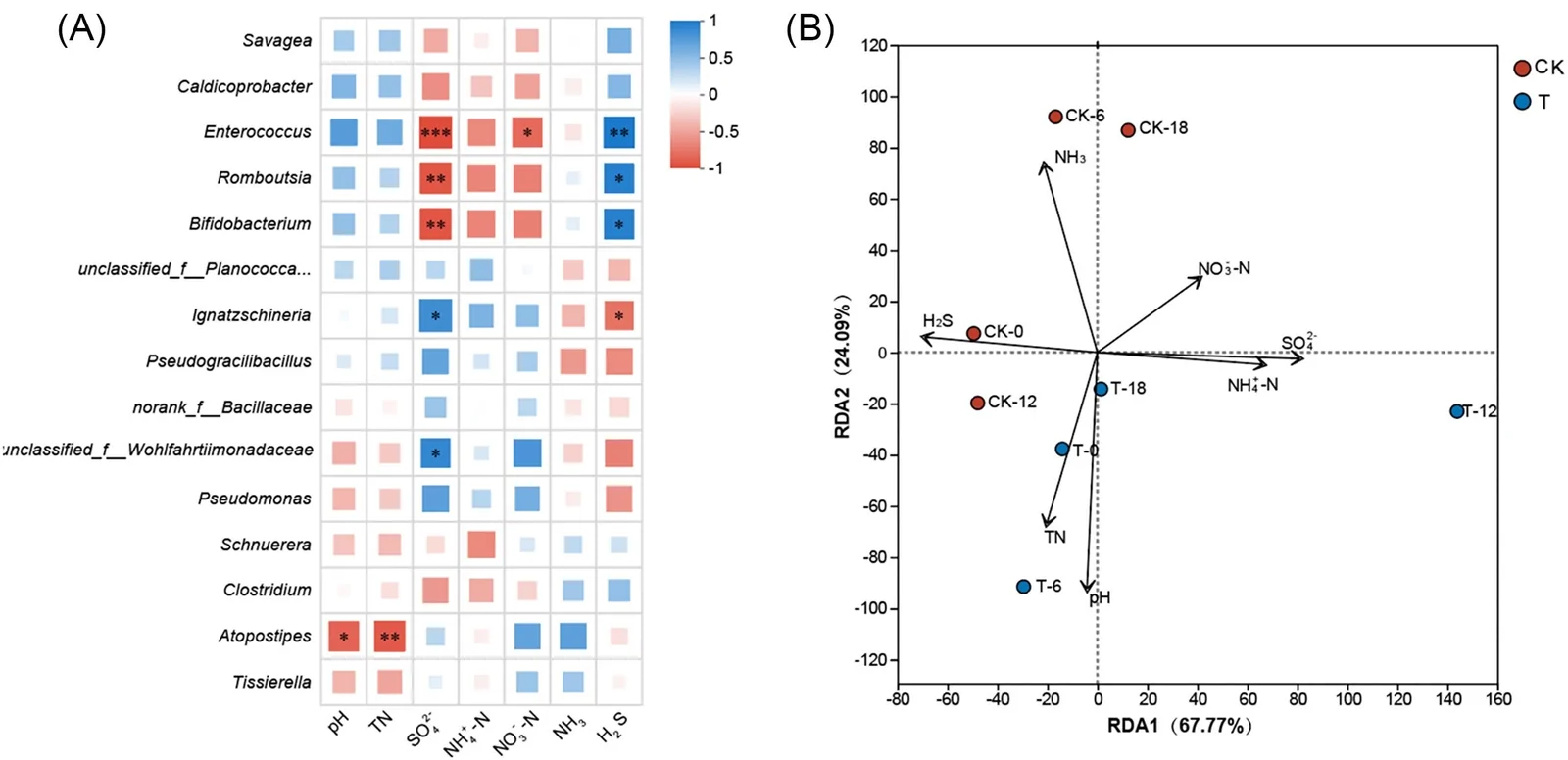
Correlation between physicochemical parameters and bacterial genera during the deodorization process: (A) Spearman correlation heatmap between environmental indicators and genus-level bacterial abundance; (B) Redundancy analysis (RDA) showing the relationship between environmental factors and microbial community structure. Note: *p* < .05 (*), *p* < .01 (**), *p* < .001 (***).

To further investigate the relationship between samples and physicochemical factors during the deodorization process, redundancy analysis (RDA) was performed, as shown in Figure 6B. The first two RDA axes explained 67.77% (RDA1) and 24.09% (RDA2) of the total variation, respectively. The ranking of environmental variables in terms of their influence on microbial community structure was as follows: pH > SO₄²⁻ > NH₃ > TN > H₂S > NH₄⁺-N > NO₃⁻-N. Specifically, NO₃⁻-N was positively correlated with NH₃ emissions, SO₄²⁻, and NH₄⁺-N, but negatively correlated with pH, TN, and H₂S emissions. This positive association between NO₃⁻-N and NH₃ emissions should not be interpreted as a direct causal effect of nitrate on ammonia volatilization. Instead, it likely reflects their co-occurrence during periods of active nitrogen turnover, when intensive ammonification increases NH₃/NH₄⁺ availability and simultaneously provides substrates for nitrification, leading to NO₃⁻-N accumulation. Therefore, the RDA result indicates covariance among nitrogen-transformation variables across samples rather than a contradiction to the role of nitrification in reducing NH₃ loss. Notably, in the CK group, H₂S and NH₃ were the environmental factors most strongly associated with microbial community structure, whereas in the T group, TN and pH were the dominant influencing factors. This shift in primary environmental drivers—from odorous gases themselves to nutrient and physicochemical parameters—suggests that the microbial consortium altered the ecological drivers shaping community composition during the deodorization process.

### Elucidation of underlying mechanisms: functional genes and metabolic pathways

Metagenomic reads were functionally annotated against the KEGG database to link community shifts with gaseous emissions. Consistent with biogeochemical theory, the nitrogen cycle encompassed assimilation, ammonification, nitrification, denitrification, anammox, and fixation (Kuypers et al., 2018). Twenty-six N-transformation genes were detected; pathway context and groupwise abundances are summarized in Figure 7A (nitrogen) and Figure 7B (sulfur).

**Figure 7:**
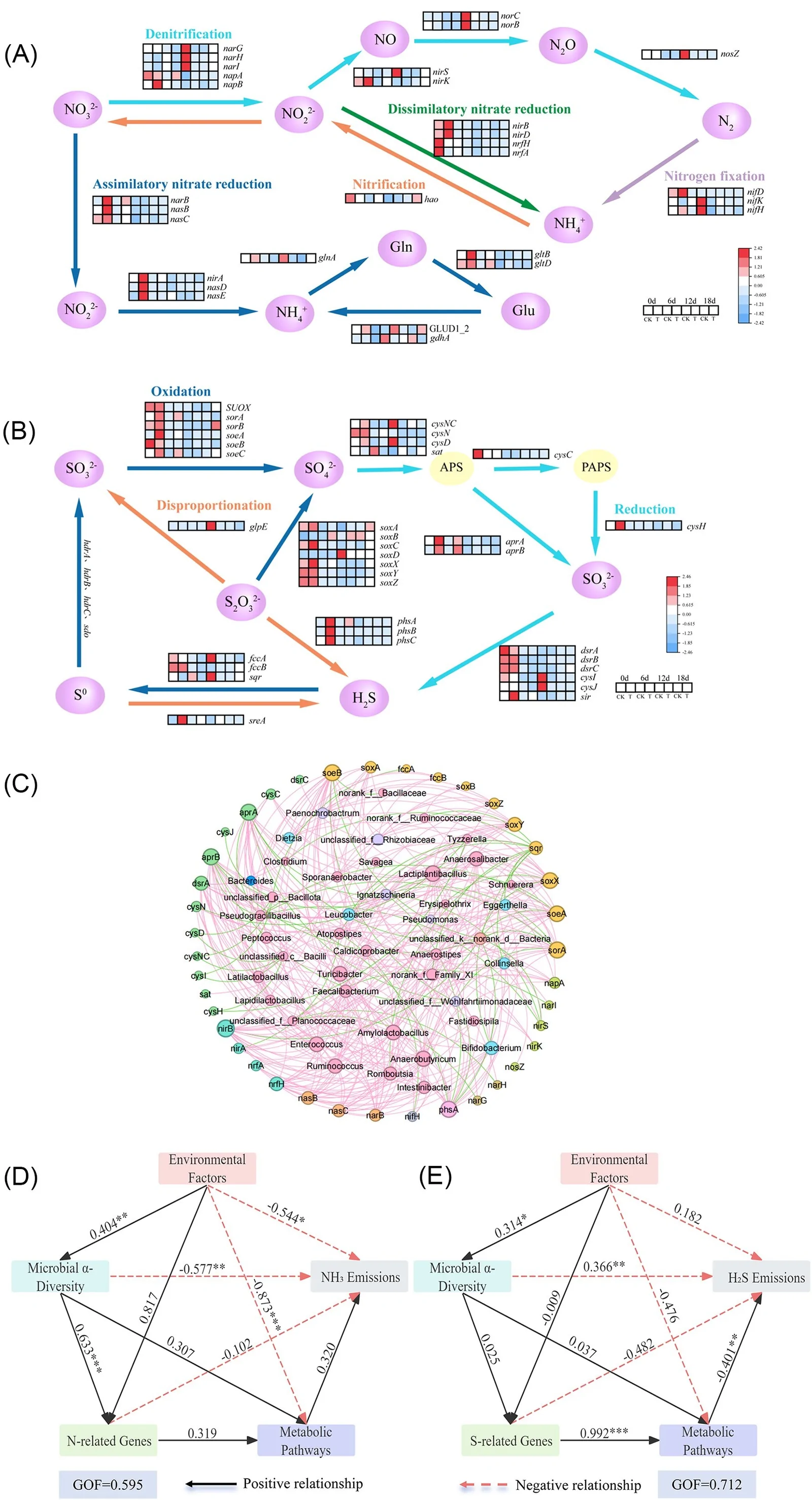
Functional mechanisms linking the composite inoculum to gas mitigation. (A) Nitrogen-cycle pathways with heatmaps of key genes (e.g., *narI, nirS, norB, nosZ, hao, glnA/gltB/gltD*). (B) Sulfur-cycle pathways with gene heatmaps (e.g., *fccA/fccB, sqr, sorA/sorB/soeC, aprA/aprB, phsB/phsC*). (C) Genus–gene co-occurrence network (nodes: genera, green = N genes, orange = S genes; edges: Spearman ρ > .6, *p* < .01). (D) PLS-PM for NH₃: standardized coefficients; dashed = negative paths; GOF shown. (E) PLS−PM for H₂S with analogous notation.

Functional genes carried by microorganisms primarily regulate nitrogen metabolism, and changes at the gene level provide a direct explanation for nitrogen transformation processes (Xiong et al., 2021). The results of this study demonstrate that during the deodorization process, key genes associated with nitrification, denitrification, and dissimilatory pathways were more abundant in the treatment group inoculated with the microbial consortium, indicating that the addition of the consortium enhanced overall nitrogen metabolism capacity. In the denitrification pathway, the genes *narI, nirS, norC, norB*, and *nosZ*—which play key roles in regulating the reduction of nitrate to nitrogen gas—were significantly upregulated in the treatment group (adjusted *p* < .05). This suggests that more nitrogen was likely transformed into N₂, thereby reducing the accumulation and emission of NH₃. In contrast, *nif* genes—associated with nitrogen fixation via nitrogenase activity and typically expressed by specific diazotrophic bacteria (Alfonzo et al., 2022)—were significantly downregulated in the treatment group compared to the control (adjusted *p* < .05). Additionally, *nirB* and *nirD*, which encode nitrite reductases involved in dissimilatory nitrate reduction, showed significantly higher expression in the control group. These findings indicate that microbial inoculation suppressed the expression of *nif, nirB*, and *nirD*, thereby limiting NH₄⁺-N accumulation and contributing to a reduction in NH₃ emission. These results align with those of Zhong et al. (2020). The *hao* gene, a well-known marker for nitrification (Wang et al., 2024a), although expressed at relatively low levels overall, was significantly upregulated in the treatment group (adjusted *p* < .05). This suggests that the microbial inoculant enhanced nitrification, promoting the conversion of NH₄⁺ into nitrate and reducing direct NH₃ release. Furthermore, genes related to amino acid synthesis, including *glnA, gltB*, and *gltD*, were annotated and found to be highly abundant, indicating active amino acid metabolic pathways within the microbial community. This implies that more nitrogen may have been incorporated into amino acids rather than being emitted as NH₃. Similar findings were reported by Zhang Mingye, who demonstrated that the addition of microbial agents increased the abundance of amino acid metabolism-related genes, such as *glnA* and *gs*, during composting (QiHang et al., 2024). In summary, microbial inoculation significantly influenced the expression of genes involved in nitrogen metabolism. The addition of the microbial consortium downregulated *nif, nirB*, and *nirD*, while upregulating *narI, nirS, norC, norB, nosZ*, and *hao*, leading to reduced NH₄⁺ formation and accumulation, enhanced nitrification and N₂ emission, and ultimately lower NH₃ release from feces. This represents a fundamental shift in nitrogen flow from volatile loss to immobilized or dinitrogen forms.

The sulfur cycle was resolved into sulfide/sulfur oxidation, dissimilatory sulfate reduction, disproportionation, and organic-S metabolism (Figure 7B). Overall sulfur-gene abundance was higher in T, indicating increased sulfur-processing capacity (Li et al., 2023). Under anaerobic conditions, the degradation of sulfur-containing proteins or amino acids, along with dissimilatory sulfate reduction, is a significant cause of malodorous sulfur compound formation and emission (Chen et al., 2023). The genes *aprA* and *aprB*, which encode key enzymes in dissimilatory sulfate reduction and catalyze the conversion of APS (adenosine-5′-phosphosulfate) into sulfite, were significantly downregulated in the treatment group (adjusted *p* < .05). This suggests that the microbial consortium may have inhibited the reduction of sulfate to sulfide, thereby reducing the production of malodorous gases such as H₂S (Wang et al., 2022d). In contrast, key genes involved in the sulfur oxidation pathway—*fccA, fccB*, and *sqr*—were upregulated in the treatment group (adjusted *p* < .05), indicating that the microbial consortium enhanced the oxidation of sulfide to sulfate and further limited the accumulation of volatile sulfur compounds. Genes involved in sulfite oxidation and electron transfer, including *sorA, sorB*, and *soeC*, were also significantly upregulated (adjusted *p* < .05), promoting the conversion of sulfite to sulfate. The gene *cysJ*, which encodes the large subunit of assimilatory sulfite reductase, catalyzes the reduction of sulfite to H₂S for use in cysteine biosynthesis. The observed downregulation of *cysJ* in the treatment group (adjusted *p* < 0.05) suggests that the microbial consortium may have reduced the assimilation of sulfur into organic forms such as cysteine. This trend, together with the upregulation of sulfur oxidation genes, supports the conclusion that the microbial consortium modulated sulfur metabolic pathways to favor oxidative over reductive and assimilatory processes, ultimately minimizing the production of volatile sulfur compounds. During sulfur disproportionation, *phsB* and *phsC*, which are involved in converting thiosulfate into sulfide and sulfite, were significantly downregulated in the treatment group (adjusted *p* < .05). This further supports the inhibitory effect of the microbial inoculant on H₂S formation via sulfur reduction pathways. In summary, the addition of the microbial deodorizing consortium downregulated *phsB, phsC, aprA*, and *aprB*, thereby suppressing sulfur disproportionation and dissimilatory sulfate reduction. Simultaneously, it upregulated *fccA, fccB, sqr, sorA, sorB*, and *soeC*, promoting sulfur oxidation reactions. These combined regulatory effects effectively reduced H₂S production, demonstrating the consortium’s potential in controlling sulfur-based odor emissions through metabolic pathway redirection.

To link taxa with function, we constructed a genus–gene co-occurrence network (Spearman ρ > 0.6, *p* < .01; Figure 7C). This network approach moves beyond simply listing correlations to reveal how the CMD fosters the assembly of integrated ‘‘functional modules’’ that collectively drive nitrogen and sulfur transformations. The network structure reveals that, within the nitrogen metabolism pathways, *Bacillus* and *Paenochrobactrum* show strong positive co-occurrence with key denitrification genes such as *narI, nirS, norB*, and *nosZ*, forming a cohesive ‘‘denitrification module.’’ This suggests that these genera may play dominant roles in N₂ production and enhance the system’s overall denitrification capacity through synergistic interactions (Wang et al., 2022a). The formation of such a module indicates that the CMD does not simply introduce isolated functions but orchestrates a multigenus coalition capable of completing the entire denitrification pathway, thereby efficiently channeling nitrogen away from NH₃ volatilization and toward harmless N₂. In addition, Pseudomonas is closely associated with genes such as *hao* and *glnA*, indicating its strong functional role in nitrification and amino acid biosynthesis. This suggests that Pseudomonas contributes significantly to both ammonia nitrogen reduction and nitrogen assimilation processes (Schmidt et al., 2022). forming a ‘‘nitrification-assimilation module.’’ Within the sulfur metabolism pathways, genera such as *Tyzzerella, Eggerthella*, and *Anaerobacillus* showed strong co-occurrence with sulfur oxidation genes, including *sqr, sorA*, and *fccA*. This reveals the assembly of a ‘‘sulfur oxidation module’’ centered on these genera, promoting efficient conversion of sulfide to sulfate (Anantharaman et al., 2018). The ecological significance of this modular structure lies in its potential for functional redundancy and stability; multiple genera contributing to the same pathway can buffer against environmental fluctuations and ensure sustained oxidative activity. Conversely, anaerobic sulfate-reducing genera such as Clostridium were negatively correlated with dissimilatory reduction genes *phsB* and *aprA*, further confirming the inhibitory effect of the consortium on sulfur-reducing pathways. This negative correlation is consistent with previous studies on microecological strategies for controlling H₂S generation (Friedrich et al., 2005) and demonstrates that suppression operates at both the taxonomic and genetic levels. The network therefore indicates that gas mitigation emerges from structure–function synergy: community reassembly activates N/S functional modules while suppressing reductive, odor-forming routes, providing a systems-level view of how the CMD engineers the fecal microbiome toward desirable metabolic outcomes.

A partial least squares path model (PLS-PM) integrated environmental factors (E: pH, NH₄⁺-N, NO₃⁻-N, SO₄²⁻), microbial α-diversity (B), functional genes (G), metabolic pathways (P), and gas emissions (N). For NH₃ (Figure 7D), environmental variables exerted a direct negative effect on emissions (E→N β ≈ −0.54, *p* < .05), while diversity imposed an indirect suppression via upregulating nitrogen-cycle genes (B→G β ≈ 0.63, *p* < .001; B→N β ≈ −0.58, *p* < .01). Prior studies have demonstrated that pH regulation constrains the activity and metabolic rate of ammonia-oxidizing bacteria, thereby affecting ammonia transformation and volatilization processes (Faust et al., 2023). Furthermore, Soratur et al. (2024) reported that enhanced microbial diversity can improve system resilience and reduce nitrogen loss through volatilization. In contrast, the H₂S model (Figure 7E) revealed that its emission was predominantly governed by sulfur-related functional genes and associated metabolic pathways. Sulfur-cycle genes showed a strong positive effect on the activation of metabolic pathways (G→P: β ≈ 0.99, *p* < .001), while the downstream pathways exerted a significant negative influence on H₂S emissions (P→N: β ≈ −0.40, *p* < .01). Although environmental factors did not directly affect H₂S release, they played an indirect regulatory role by influencing microbial diversity and gene expression (E→B, E→G). This regulatory scheme is consistent with previous findings on the key roles of *dsrA* and *aprA* in H₂S production, as well as the modulation of sulfur metabolism by SO₄²⁻ availability (Blazejak and Schippers, 2011; Ci et al., 2022). It also aligns with reports highlighting the role of sox genes in cysteine degradation pathways under nonsulfate conditions (Nguyen-tiêt et al., 2024). Although both gas models share the path structure E→B→G→P→N, dominant levers differ: NH₃ suppression is mainly diversity-mediated through nitrogen functional genes, whereas H₂S control is gene- and pathway-centric within the sulfur module. This differential regulatory architecture provides a mechanistic explanation for why the CMD achieves simultaneous removal of both gases through distinct but complementary pathways—a key insight for designing targeted bioaugmentation strategies.

From a broader perspective, the integrated multiomics and modeling approach employed here reveals that the CMD functions as a microbial ecosystem engineer. It does not merely add new metabolic capabilities but systematically rewires the existing microbial network—activating oxidative functional modules, suppressing reductive pathways, and shifting the primary environmental drivers shaping community structure. This systems-level understanding of bioaugmentation represents a significant advance over previous studies that focused solely on removal efficiencies or isolated gene expression changes. The mechanistic insight gained here—that successful intervention requires not just introducing functional strains but orchestrating their integration into coherent metabolic modules—provides a theoretical foundation for designing more predictable and robust microbial deodorization strategies for complex waste systems.

## Conclusions

This study developed, validated, and elucidated the mechanism of a CMD for sustainable odor and exposure control in decentralized rural toilets. The principal findings are as follows:

1. Five efficient bacterial strains were successfully screened from activated sludge and assembled into an optimal CMD with a 1:1:1:1:1 ratio determined by orthogonal testing. Laboratory validation confirmed its synergistic deodorization capacity, achieving significant removal rates of 82.1% for NH₃ and 86.1% for H₂S.
2. Field application across three typical rural toilet types in Hebei Province demonstrated robust and rapid real-world efficacy. Peak removal rates reached 79.43% for NH₃ and 80.83% for H₂S. Notably, the triple-tank septic system exhibited excellent performance stability, with gas suppression effectively maintained over the 24-week monitoring period at levels significantly below preapplication baselines (*p* < .05 at all post-application time points), confirming sustained functional colonization.
3. The CMD addition significantly modulated the physicochemical environment. It helped maintain a relatively stable, neutral-to-alkaline pH, reduced NH₄⁺-N, increased NO₃⁻-N, and decreased total nitrogen loss by 14.8%. The concurrent significant rise in SO₄²⁻ concentration indicated a metabolic shift toward sulfur oxidation.
4. Integrated metagenomic and PLS-PM analyses revealed the underlying microbial mechanism. The CMD systematically rewired the fecal microbiome by upregulating key genes involved in nitrogen/sulfur oxidation (e.g., *narI, nosZ, sqr*) while suppressing those for reductive pathways (e.g., *aprA, nif*), thereby blocking odorant generation at the metabolic source. Co-occurrence network analysis further elucidated the assembly of functional modules—such as a ‘‘denitrification module’’ centered on *Bacillus* and a ‘‘sulfur oxidation module’’ involving *Tyzzerella*—demonstrating that the CMD fosters synergistic interactions between functional genera and genetic pathways. PLS-PM revealed distinct regulatory architectures: NH₃ suppression was primarily diversity-mediated through nitrogen functional genes, whereas H₂S control was gene- and pathway-centric within the sulfur module.

This work completes a full ‘‘construction–validation–mechanism’’ evidence chain, providing a novel, systems–level understanding of how a defined microbial consortium functions as an ecological engineer to redirect N and S metabolic fluxes at the genetic and community levels. It transitions an *in situ* microbial deodorization strategy from a laboratory concept to a field–verified, practical intervention. The CMD offers a low–cost, eco–friendly solution to directly mitigate hazardous gas exposure at the household level, providing clear co–benefits for public health, sanitation acceptance, and environmental sustainability in rural communities. Future work should focus on validating this strategy across diverse climatic and geographic regions, developing cost–effective formulation and application protocols, and conducting comprehensive ecological safety assessments to facilitate its widespread and sustainable implementation.

## Supplementary Material

kuag024_Supplemental_File

## Data Availability

Data will be made available on request.
