# Supplementary material for: Field-validated microbial deodorant reduces hazardous gas exposure and improves air quality in rural household toilets
Source: J Ind Microbiol Biotechnol. 2026 Oct 14;53:kuag024. doi: 10.1093/jimb/kuag024 (PMC13662806; doi:10.1093/jimb/kuag024)
Supplement: Supplementary file 1 [file kuag024_supplemental_file.docx]

# 1. Supplementary Methods

## *S1.1. Media compositions, enrichment procedures, and primary screening analytics*

To enrich deodorizing microorganisms targeting nitrogen and sulfur transformations, two selective liquid media were employed. The ammonia-enrichment medium consisted of (g/L): (NH₄)₂SO₄ 1.0, KH₂PO₄ 1.5, K₂HPO₄ 1.5, MgSO₄·7H₂O 0.2, CaCl₂ 0.05, trace elements, adjusted to pH 7.5. The sulfur-removal enrichment medium contained (g/L): Na₂S·9H₂O 0.2, NaHCO₃ 2.5, KH₂PO₄ 1.5, K₂HPO₄ 1.5, MgSO₄·7H₂O 0.2 (pH 7.2). Activated sludge (10 g, secondary sedimentation tank) was suspended in 90 mL sterile water and shaken at 30 °C (150 rpm) for 30 min to obtain a homogeneous suspension. Aliquots of the suspension were inoculated into both enrichment media and subcultured every five days under identical incubation conditions to selectively promote organisms involved in the target pathways.

Following enrichment, cultures were subjected to tenfold serial dilutions and spread on corresponding selective agar plates. Plates were incubated at 30 °C for approximately three days. Morphologically distinct single colonies were streak-purified, examined microscopically for preliminary classification, and preserved on slants at 4 °C. For primary screening, seed cultures were prepared in beef extract–peptone broth and inoculated (2% v/w) into ammonia- and sulfur-removal screening media. Incubations were conducted at 30 °C with shaking at 150 rpm for 48 h (ammonia removal) or 8 h (sulfur removal), respectively. Ammonia removal was quantified by determining residual NH₄⁺-N using the Nessler’s reagent colorimetric method, while sulfide removal efficiency was determined by the p-amino dimethylaniline (PADA) method assessing S²⁻ in the culture supernatant. Each assay included a sterile blank and three biological replicates. Where applicable, sampling, reagent blanking, and wavelength settings followed the cited standard protocols to ensure analytical comparability with prior studies.

Analytics note. For routine physicochemical analyses related to S1 (pH, TN, NH₄⁺-N, NO₃⁻-N, SO₄²⁻), procedures and calibrations adhered to standard methods . TN was measured using an elemental analyzer; where matrix effects were suspected, dilutions were performed to maintain absorbance within the linear range.

## *S1.2. Gas-release simulation devices and quantification for NH₃ and H₂S*

To simulate NH₃ capture, 200 g of secondary-screening medium inoculated with candidate strains was placed in a PVC cylindrical column (length 20 cm, inner diameter 16 cm). Two circular sponges (thickness 2 cm; diameter matching the column) were stacked within the column; each sponge was saturated with 20 mL glycerol phosphate solution. The upper sponge served primarily as a barrier to external air interference, while the lower sponge captured NH₃ released during microbial activity. The lower sponge was replaced every 24 h. Ammonium was extracted from the recovered sponge by shaking in 1 mol L⁻¹ KCl solution at 160 rpm for 1 h, followed by colorimetric quantification of NH₄⁺-N using the sodium hypochlorite–salicylic acid method. The overall ammonia removal efficiency was computed based on the cumulative NH₄⁺-N extracted from the absorber relative to the control.

For H₂S, 200 g of inoculated screening medium was placed in a 3 L beaker containing 20 mL of a designated H₂S absorption solution. The beaker was sealed with plastic film to create a closed fermentation microenvironment. The absorption solution was replaced at 24 h intervals, and dissolved sulfide concentrations were determined by the methylene blue spectrophotometric method. A blank control consisting of sterile medium was included under identical incubation conditions. All treatments were conducted in triplicate. Supplementary Fig. S1 illustrates the NH₃ capture configuration and sampling workflow.

Fig. S1. Experimental setup for secondary screening of ammonia-removing strains

## *S1.3. Strain identification, antagonism testing, and growth characterization*

Strains with superior deodorization performance in S1.2 were subjected to an expanded identification workflow including colony morphology on solid media, physiological and biochemical testing, and molecular identification by 16S rRNA gene sequencing. Biochemical assays followed standard protocols (e.g., oxidase, catalase, citrate utilization, starch hydrolysis, and carbohydrate fermentation/oxidation profiles). Taxonomic assignment was performed by aligning 16S sequences against the EzBioCloud database; phylogenetic trees were constructed in MEGA v11.0 with bootstrap analysis to evaluate node stability.

Inter-strain compatibility was assessed by the paper disc diffusion method. Sterile filter-paper discs loaded with candidate strains were placed on agar plates pre-inoculated with potential partners; plates were incubated at 30 °C for 48 h, and inhibition zones were recorded to infer antagonism. The resulting compatibility matrix guided the subsequent consortium design. Raw outcomes, morphology, growth curves, biochemical profiles, phylogenies, and compatibility matrices are presented in Supplementary Results (Fig. S2–S4; Table S2–S4) .

## *S1.4. Construction of microbial consortia and deodorization testing workflow*

Following S3, strains without detectable antagonism were considered for consortium assembly. An orthogonal design Lₘ(2ⁿ) was adopted with five bacterial strains as factors and inoculated/non-inoculated conditions as two levels to efficiently explore interactions (Table 1). Candidate consortia were prepared by mixing log-phase cultures at predefined proportions and inoculated into fecal matrices for performance screening.

For bench-scale testing, 200 g of feces was placed in an appropriate vessel and inoculated at 10% (v/w) with each candidate consortium; sterile medium at equal volume served as the control. NH₃ was monitored using a portable ammonia gas detector, and H₂S was absorbed in a designated solution prior to spectrophotometric determination. The optimal consortium was then subjected to validation in a 3 kg system. Specifically, 3 kg of fresh feces was thoroughly mixed with 10% (v/w) consortium (treatment, T) or equal volume sterile medium (control, CK) and placed into a sealed foam box (50 cm × 25 cm × 40 cm). A 100 mL beaker containing 50 mL H₂S absorption solution was positioned inside the box. Boxes were maintained at room temperature, and NH₃ and H₂S measurements were taken daily at 16:00. Fecal samples were collected by a five-point method; each composite sample was split into three replicates and stored at −80 °C for physicochemical analysis and metagenomic sequencing. This validation protocol ensured comparability between bench- and validation-scale outcomes while maintaining consistent sampling, storage and analytical stringency.

## *S1.5. Metagenomic DNA extraction, library preparation, sequencing, and bioinformatics*

DNA was extracted from fecal samples using the E.Z.N.A.® Soil DNA Kit (Omega Bio-Tek, USA). DNA purity and concentration were assessed by NanoDrop and Qubit fluorometry, while integrity was verified on 1% agarose gels. Sequencing libraries were prepared using a standard Illumina workflow (fragmentation, end repair, adapter ligation, PCR amplification, and quantification) and sequenced on an Illumina NextSeq 2000 platform in paired-end mode. All laboratory procedures (extraction, library construction, sequencing) were performed by Shanghai Meiji Biomedical Technology Co., Ltd., with samples processed in a single batch to minimize batch effects.

Raw paired-end reads underwent adapter removal and quality trimming using fastp. High-quality reads were assembled de novo with MEGAHIT to generate contigs. Open reading frames (ORFs) were predicted using Prodigal (meta mode). To construct a non-redundant gene catalog, predicted genes from all samples were clustered at 90% identity with CD-HIT, and the representative sequence of each cluster was retained. High-quality reads from each sample were mapped to the non-redundant catalog using SOAPaligner; gene abundance profiles were generated with appropriate normalization (e.g., CPM/TPM) to allow cross-sample comparisons. Functional annotation followed KEGG orthology to identify genes associated with nitrogen and sulfur cycling. Community diversity indices and rarefaction analyses were computed using standard R workflows; statistical visualization was performed in OriginPro 2024.

# 2. Supplementary Results：

## *S2.1. Morphological and Physiological Characteristics of Candidate Deodorizing Strains*

The colony morphologies of the five candidate strains are shown in Fig. S2. Strain N-4 formed grayish-white, opaque colonies with smooth surfaces and regular margins; N-7 formed golden-yellow, opaque, smooth, circular colonies; N-12 formed pale yellow, opaque colonies with smooth, moist surfaces and irregular margins; S-6 formed yellow, opaque colonies with wrinkled surfaces and irregular edges; and S-13 formed yellow, opaque colonies with smooth, moist surfaces. Preliminary morphological observations and Gram staining were performed on these five candidate strains for the composite microbial agent. The physiological and biochemical characteristics of each strain are summarized in Table S2.

**Fig. S2.** Colony morphology of the highly efficient deodorizing strains

**Table S2.** Physiological and Biochemical Characteristics of Candidate Deodorizing Strains

| **Test Item** | **N-4** | **N-7** | **N-12** | **S-6** | **S-13** |
| --- | --- | --- | --- | --- | --- |
| Cell Morphology | Rod-shaped | Rod-shaped | Rod-shaped | Rod-shaped | Rod-shaped |
| Gram Staining | – | + | + | – | – |
| Lysozyme | + | – | + | – | – |
| Mannitol | – | – | – | – | – |
| Glucose | – | – | – | + | – |
| Starch Hydrolysis | – | – | – | + | – |
| Nitrate Reduction | – | + | – | – | – |
| Voges-Proskauer (VP) Test | – | + | – | – | – |
| Gelatin Hydrolysis | – | – | – | – | – |
| Citrate Utilization | + | – | – | + | + |
| 3% Hydrogen Peroxide | + | + | – | + | + |

Note: “+” indicates positive reaction; “–” indicates negative reaction.

## *S2.2. Molecular identification of deodorizing strains*

Traditional taxonomic methods based on phenotypic characteristics (such as colony morphology and physiological-biochemical tests) have certain limitations and potential biases in strain identification. In contrast, molecular biological identification provides a more accurate and reliable tool for taxonomic classification. The gene sequence data provided by the sequencing facility were uploaded to the EzBioCloud database for alignment. The results are presented in Table 1. Phylogenetic trees were constructed using the neighbor-joining (NJ) method in MEGA version 11.0. Strain N-4 (S3(a)) clustered in the same branch with Alcaligenes faecalis subsp. phenolicus DSM 16503ᵀ, sharing 99.86% sequence similarity. Strain N-7 (S3(b)) clustered with Arthrobacter luteolus NBRC 107841ᵀ, showing 99.57% similarity. Strain N-12 (S3(c)) clustered with Lysinibacillus fusiformis NBRC 15717ᵀ, with 99.93% similarity. Strain S-6 (S3(d)) clustered with Stutzerimonas balearica DSM 6083ᵀ, showing 99.71% similarity. Strain S-13 (S3(e)) clustered with Pseudomonas guariconensis LMG 27394ᵀ, with 99.43% similarity.

Fig. S3. Phylogenetic tree of highly effective deodorizing bacteria

## *S2.3. Growth Curves of the Strains*

The growth curves of the five strains are shown in Fig. S4. All strains reached the stable phase within ~15 h; the intrinsic growth rate ranked N-7 > S-13 > N-12 > S-6 > N-4, informing subsequent inoculation scheduling.

Fig. S4. Growth curves of the deodorizing strains.

## *S2.4. Antagonism Experiment*

Antagonism tests were conducted among the five strains N-4, N-7, N-12, S-6, and S-13. The results are shown in Supplementary Table S4. The experimental data indicate that no antagonistic interactions occurred between any of the strains; therefore, these strains can be randomly combined for the construction of the composite deodorizing microbial agent.

**Table S4.** Results of antagonism experiments between bacterial strains

| Strain | N-4 | N-7 | N-12 | S-6 | S-13 |
| --- | --- | --- | --- | --- | --- |
| N-4 | \ | – | – | – | – |
| N-7 | – | \ | – | – | – |
| N-12 | – | – | \ | – | – |
| S-6 | – | – | – | \ | – |
| S-13 | – | – | – | – | \ |

Note: “+” indicates antagonism; “–” indicates no antagonism; “\” indicates self-comparison (not applicable).

## *S2.5. Species Rarefaction Curve*

Rarefaction analysis based on the Sobs index (Fig. S5) approached a plateau, indicating near-saturation sequencing depth. The minimal increase in observed OTUs with additional reads suggests adequate coverage and reliable representation of the underlying bacterial community during deodorization.

Fig. S5. Bacterial dilution curve

## *S2.6. Core Microbial Community Analysis at Different Time Points*

Fig. S6 presents Venn diagrams for both groups over time. Across eight samples, 164 OTUs were shared. Unique OTUs (CK vs T) numbered 65 vs 74 (day 0), 76 vs 71 (day 6), 70 vs 25 (day 12), and 78 vs 68 (day 18). These patterns indicate that inoculation altered community composition relative to the uninoculated control at identical time points, and that the structures of both communities evolved markedly over time—consistent with prior observations that distinct substrates select distinct core populations during deodorization^[1-3]^.

Fig. S6. Bacterial Venn diagrams showing OTU distribution at different time points.

**Reference**

[1]	Wang S, Xu Z, Xu X, Gao F, et al. Effects of two strains of thermophilic nitrogen-fixing bacteria on nitrogen loss mitigation in cow dung compost [J]. Bioresource Technology, 2024, 400: 130681.

[2]	Wang Q, Liu Y, Yang S, Wang Y, et al. The treatment performance and the removal mechanism of pollutants in vertical flow constructed wetlands combined with magnetic field [J]. Journal of Water Process Engineering, 2025, 71: 107311.

[3]	Ao Q, Ni Z, Su L, Zhao H, et al. Effect of iron-carbon microelectrolysis and magnetite on biological nitrogen removal: Analysis of microbial communities, functional genes, and mechanisms [J]. Environmental Research, 2025, 274: 121229.
